# The Role of the GH/IGF1 Axis on the Development of MAFLD in Pediatric Patients with Obesity

**DOI:** 10.3390/metabo12121221

**Published:** 2022-12-05

**Authors:** Antonella Mosca, Luca Della Volpe, Anna Alisi, Nadia Panera, Giuseppe Maggiore, Andrea Vania

**Affiliations:** 1 Hepatogastroenterology, Nutrition, Digestive Endoscopy and Liver Transplant Unit, Bambino Gesù Children’s Hospital, IRCCS, 00165 Rome, Italy; 2 Pathology Unit, Department of Diagnostic and Laboratory Medicine, Bambino Gesù Children’s Hospital, IRCCS, 00165 Rome, Italy; 3 Independent Researcher, 00162 Rome, Italy

**Keywords:** GH, nonalcoholic fatty liver disease, fibrosis, IGF1

## Abstract

The anomalies of the Growth Hormone (GH)/Insulin-like Growth Factor-1 (IGF1) axis are associated with a higher prevalence of Metabolic Associated Fatty Liver Disease (MAFLD) and with a more rapid progression towards fibrosis, cirrhosis, and end-stage liver disease. A total of 191 adolescents with obesity [12–18 years] were consecutively enrolled between January 2014 and December 2020 and underwent liver biopsy to diagnose MAFLD severity. In all patients GH, IGF1 and Insulin-like Growth Factor-Binding Protein 3 (IGFBP3) were measured. Patients with inflammation and ballooning have significantly lower values of GH and IGF1 than those without (GH: 5.4 vs. 7.5 ng/mL; IGF1 245 vs. 284 ng/mL, *p* < 0.05). GH and IGF1 were also negatively correlated with fibrosis’ degree (r = −0.51, *p* = 0.001, and r = −0.45, *p* = 0.001, respectively). Only GH correlated with TNF-a (r = −0.29, *p* = 0.04) and lobular inflammation (r = −0.36, *p* = 0.02). At multivariate regression, both GH and IGF1 values, after adjustment for age, sex and BMI, were negatively associated with HOMA-IR but above all with fibrosis (GH→β = −2.3, *p* = 0.001, IGF1→β = −2.8, *p* = 0.001). Even in the pediatric population, a reduction of GH input in the liver directly promotes development of de novo hepatic lipogenesis, steatosis, fibrosis and inflammation. The possible role of recombinant GH administration in adolescents with obesity and severe MAFLD deserves to be studied.

## 1. Introduction

Metabolic Associated Fatty Liver Disease (MAFLD) is one of the main complications of obesity and has become the most frequent liver disease in children and adolescents. Furthermore, obesity is associated with a reduced secretion of Growth Hormone (GH) with consequent reduced secretion of Insulin-like Growth Factor-1 (IGF1) from the liver [1]. Recent pre-clinical studies suggest that the anomalies of the GH/IGF1 axis are associated with a higher prevalence of steatosis, with a more rapid progression towards non-alcoholic steatohepatitis (NASH), cirrhosis and end-stage liver disease [2].

GH and IGF1 are important regulators of glucose and lipid metabolism and have anti-inflammatory and anti-fibrotic effects [3]. GH and IGF1 have opposite effects on glycemic metabolism: GH acts as a counter-regulating hormone, increasing blood sugar levels by promoting hepatic gluconeogenesis and glycogenolysis, and favoring the release of glucose from cells, while IGF1 exerts hypoglycemic effects, favoring the intracellular absorption of glucose and the accumulation of hepatic glycogen, given its structural homology with insulin, and inhibiting GH secretion via negative feedback [2,3]. Diverging effects are also observed on lipid metabolism in the adipose tissue, where GH promotes lipolysis while IGF1 promotes lipogenesis [2].

Due to these biological actions, in the liver the deficiency of GH and IGF1 favors the development of MAFLD, respectively, by increasing the accumulation of triglycerides in hepatocytes and by causing the development of insulin resistance [4,5]. In a recent study, the specific deletion of the hepatocyte GH receptor in mouse models was associated with a fourfold increase in circulating GH levels and strong suppression of IGF1 levels. Phenotypically, these mice exhibited insulin resistance, glucose intolerance, fatty liver and increased free fatty acid levels [6]. Fusco et al. found that in adult humans with obesity the response to the arginine release test, assessed upon circulating levels of GH, was reduced in MAFLD compared to controls [7]. Similarly, another study has shown an association between reduced GH levels and increased intrahepatic lipids in adult women with obesity [8]. Ichikawa et al. also reported that serum GH was inversely associated with MAFLD in adult subjects with obesity, but a GH stimulation test was not performed in the study [4]. Similarly to IGF1, IGF-binding proteins (IGFBPs) are also synthesized by the liver in response to GH. IGFBPs in serum bind IGF1 to regulate its action but also have independent biological actions [9]. A recent analysis suggests that circulating levels of IGFBPs increase in the developmental stage of liver fibrosis [10], while other studies suggest that MAFLD correlates with high circulating levels of IGFBP-1, IGFBP-2 and IGFBP-3 [11,12,13,14,15]. An additional protein with less binding for IGF1, called IGFBP1 or IGFBP-7, has recently emerged as a marker of insulin resistance [16] and MAFLD [17]. Based on these data, the administration of recombinant GH could therefore significantly improve steatosis, inflammation, and hepatic fibrosis in patients with either steatosis or NASH [2]. In fact, the administration of GH reduces oxidative and inflammatory mediators such as the Tumor Necrosis Factor (TNF) and acute phase proteins (ultra-sensitive C reactive protein) in the liver [18]. In studies based on animal models of liver cirrhosis, IGF1 has also been shown to have hepatoprotective effects and appears to reduce oxidative stress, insulin resistance, hepatocellular apoptosis and fibrogenesis [19]. A number of preclinical and clinical studies on animal models and adult patients confirms the correlation between GH and IGF1 levels in the development of MAFLD; At the same time, we know little in this regard about adolescents, who constitute an independent clinical and biochemical model, due to pubertal development. For these reasons, we conducted a study in a large cohort of adolescent patients with obesity and no hypothalamic-pituitary disease, aimed to confirm a positive relationship between relative GH deficiency and MAFLD, and to show, for the first time in a pediatric study, a correlation between serum levels of GH and IGF1 with hepatic histology. We have confidence that this study will help to understand how relative alterations of the GH/IGF1 axis can influence clinical deterioration and liver damage progression in patients with MAFLD.

## 2. Materials and Methods

We enrolled 191 adolescents, aged 12 to 18 years, sent to the Hepatology Department of the “Bambino Gesù” Children’s Hospital of Rome from January 2014 to December 2020, who had ultrasound imaging of hepatic steatosis and agreed to undergo liver biopsy to evaluate steatosis’ severity in accordance with ESPGHAN guidelines [20]. Patients were divided by Tanner’s stage and gender, and all were affected by obesity, with a Body Mass Index (BMI) ≥90–95th percentile. BMI and Waist Circumference percentile (WC) were calculated on the anthropometric data collected at the time of diagnosis. All patients were tested for secondary causes of fatty liver disease (e.g., Wilson’s disease, α-1-antitrypsin deficiency, viral hepatitis, autoimmune hepatitis, endocrinological, genetic and metabolic diseases, celiac disease, alcohol and/or drug use) [20,21]. Adolescents with pituitary disorders or with GH deficiency were excluded. All were Caucasians of Italian descent. The study was conducted according to the rules of the Declaration of Helsinki. Venous blood samples were collected after fasting for at least 8 h. Serum liver enzymes (aspartate aminotransferase, AST, alanine aminotransferase, ALT, and gamma-glutamyl transferase, GGT), lipids (total cholesterol, high density lipoprotein-cholesterol, HDL, low density lipoprotein-cholesterol, LDL, and triglycerides), fasting glucose, fasting insulin levels, GH, IGF1 and IGFBP-3 binding proteins were measured in all patients at the Central Laboratory of the “Bambino Gesù” Children’s Hospital. GH levels were standardized to 8 ng/mL [22,23]. In our laboratory, GH levels are defined using the ECLIA method (electrochemiluminescence); The reference values are <8.05 ng/mL for females and <10.8 ng/mL for males in the population between 10 and 18 years. We used a unique cut-off of 8 ng/mL in the whole population to define a significant deficit. The IGF1 levels were normalized for Tanner stage (I = 71.4–394, II = 122–508, III = 164–545, IV = 174–480, V = 169–400 ng/mL), and were dosed using a chemiluminescent sandwich immunoassay (CLIA). The Insulin-like Growth Factor-Binding Protein 3 (IGFBP-3) levels were considered normal for a range of 3.5–10 µg/mL, as per the standards of our analysis laboratory. The model score homeostasis assessment (HOMA-IR) was used for insulin resistance estimation, with a value > 2.5 considered as a marker of insulin resistance [24]. Liver biopsies were performed, after the consent of parents and patients was obtained, using an 18-gauge automatic biopsy needle, general anesthesia, and guided ultrasound. The histological features of fatty liver disease were steatosis, portal and lobular inflammation, balloon hepatocytes, and fibrosis. Histological analysis was performed by a single pathologist blinded to clinical and laboratory data. All samples were analyzed immediately after collection, as were all of the laboratory parameters. Steatohepatitis was characterized by the scoring system (NAS) developed by the National Institutes of Health NASH Clinical Research Network [25]. Hepatic steatosis was classified on four degrees: 0 = steatosis involving less than 5% of hepatocytes, 1 = involving steatosis = 33% of hepatocytes, 2 = involving steatosis = 33–66% and 3 = steatosis in 66% of hepatocytes. Lobular and portal inflammation were classified on four degrees: 0 = none, 1 = mild, 2 = moderate, and 3 = severe. Balloon hepatocytes were graded on three points: 0 = no balloon cells, 1 = few balloon cells, and 2 = many/prominent balloon cells. Finally, hepatic fibrosis was quantified using a five-point grading: 0 = fibrosis; 1 = perisinusoidal or periportal fibrosis, with (1a) mild, zone 3, perisinusoidal; (1b) moderate, zone 3, perisinusoidal; And (1c) portal/periportal; 2 = perisinusoidal/periportal portal fibrosis; 3 = fibrous bridge; 4 = cirrhosis [26].

Plasma levels of TNF-α and IL-6 were measured by using commercially available ELISA kits, according to the manufacturer’s instructions. In details: (i) TNF-α levels were measured by Human TNF-α ELISA Ready-SET-Go (Affymetrix eBioscience; Catalog. Number: 88–7346; San Diego, CA, USA), the detection range of the kit is 4–500 pg/mL; (ii) IL-6 levels were measured by Human IL-6 ELISA Ready-SET-Go (Affymetrix eBioscience; Catalog. Number: 88–7066; San Diego, CA, USA), the detection range of the kit is 2–200 pg/mL.

## 3. Statistical Analysis

Data are expressed as mean and SD, or as medians and interquartile intervals (IQRs), or frequencies. Differences in clinical variables were tested by the exact Fischer test for categorical variables, by unidirectional ANOVA for normally distributed continuous variables, and by the Kruskal Wallis’s test for non-normally distributed continuous variables.

Pearson and Spearman correlation coefficients were calculated to examine invariable linear associations of plasma levels of GH, IGF1 and IGFBP-3 with liver fibrosis values, metabolic parameters and cytokines. Subsequently, a multivariate linear regression modeling analysis was used to verify the independence of association between these three circulating biomarkers as key exposures, with metabolic parameters and histological characteristics as the key outcome (included as a continuous measure), after adjusting for potential confounding factors, such as age, gender, and BMI. Covariates included in all multivariable regression models were selected as potential confounders based on their significance in univariable regression analyses or on their biological plausibility. A *p* < 0.05 was considered statistically significant. Statistical analyses were performed using Medcalc software, version 20.014 (MedCalc Software Ltd., Ostend, Belgium).

## 4. Results

A total of 191 adolescents (mean age 12.9 ± 2.1 years), 87 (45%) males and 93 (55%) females, underwent liver biopsy for MAFLD between 2015 and 2022 at the Hepatology Unit of the “Bambino Gesù” Children’s Hospital. The characteristics of the population are shown in Table 1.

The GH (7.8, IQR 5.8–9 ng/mL) values as well as the IGF1 (266, IQR 198–345 ng/mL) and IGFBP-3 (2.34, IQR 1.5–3.2 ng/mL) values were appropriate for the age of patients, especially considering the dependence on pubertal stage for IGF1and GH. Mean values of BMI and WC, as well as mean values of transaminases and HOMA-IR, were high for age.

Moreover, after dividing our population by Tanner’s stage and sex, there were no differences for values of GH, IGF1 and IGFB3, as well as for metabolic syndrome and cytokine parameters (Table 2).

**Figure 1:**
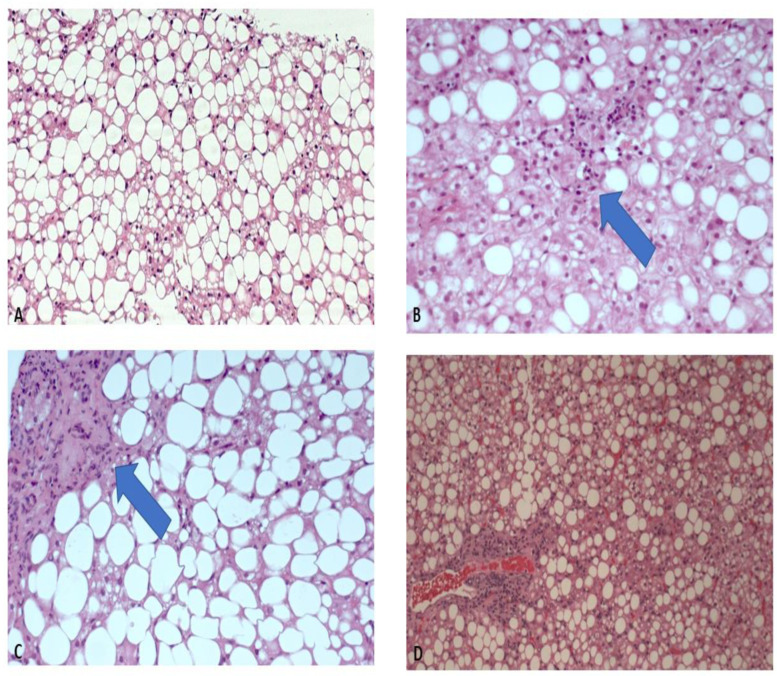
Histologic features. (**A**) Simple steatosis, without inflammation. (**B**) Grade 2 steatosis, with initial inflammation. (**C**) Grade 3 steatosis, with lobular and portal fibrosis. (**D**) Steatohepatitis with fibrosis (Hematoxylin and Eosin, 20×).

As for the histologic features, in our population 46% of the patients (89) had a diagnosis of steatohepatitis on histology (NAS > 4), and 70% had fibrosis > 1 (Table 3) (Figure 1).

In our population, adolescents with steatohepatitis had a significantly higher BMI than those without steatohepatitis. Furthermore, all our adolescents with steatohepatitis had significantly (*p* < 0.05) higher metabolic syndrome parameters (triglycerides, HOMA-IR, uric acid and basal glucose), but also significantly higher ALT and AST values (ALT 75 IQR 39–90 vs. 49, IQR 24–66 U/L; AST 50 IQR 33–62 vs. 37 IQR 25–45 U/L). Moreover, the steatohepatitis group had significantly high values of IL-6 (35 IQR 15–39 vs. 25 IQR 22–35.5) and TNF-a (72 IQR 12–72 vs. 44 IQR 7.5–44), *p* < 0.05.

Regarding GH values, patients with steatohepatitis had significantly lower values than those without such condition (5.4 IQR 4–6.8 vs. 7.5 IQR 6–8.2 ng/mL), as well as lower IGF1 (245 IQR 150–310 vs. 284 IQR 208–345 ng/mL) and IGFBP-3 values (2 IQR 1.4–2.8 vs. 2.6 IQR 1.6–3.4 µg/mL) (Table 4).

**Figure 2:**
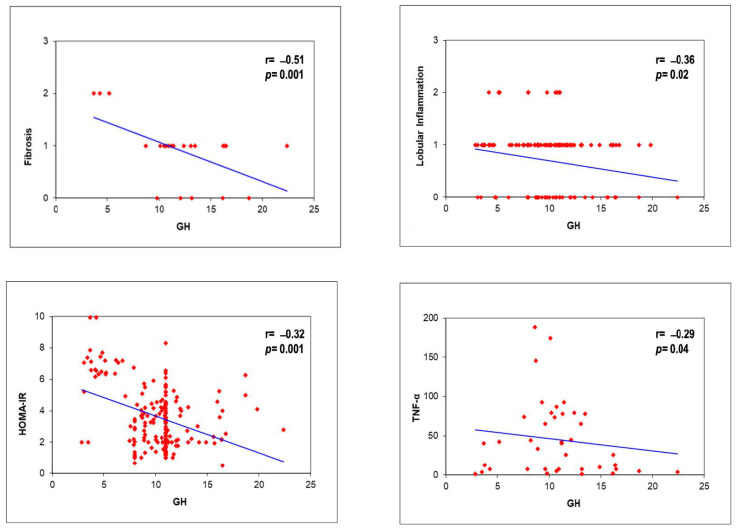
GH correlations with histology, HOMA-IR, and TNF-a.

GH levels in our population were negatively correlated with insulin (r = −0.21, *p* = 0.005) and HOMA-IR (r = −0.32, *p* = 0.0001), but especially with fibrosis (r = −0.51, *p* = 0.001) and lobular inflammation (r = −0.36, *p* = 0.02) at liver biopsy. Moreover, the GH negatively correlated with TNF-a (r = −0.29, *p* = 0.04), but not with IL-6. IGF1 levels negatively correlated with BMI (r = −0.15, *p* = 0.03), glucose (r = −0.28, *p* = 0.0001), HOMA-IR (r = −0.35, *p* = 0.0001), total-cholesterol (r = −0.38, *p* = 0.001), and fibrosis (r = −0.45, *p* = 0.001) but not with other histological parameters and cytokines. Finally, IGFBP-3 values correlated negatively with BMI (r = −0.19, *p* = 0.02), glucose (r = −0.23, *p* = 0.02), HOMA-IR (r = −0.27, *p* = 0.003), total-cholesterol (r = −0.21, *p* = 0.005), as well as with fibrosis (r = −0.45, *p* = 0.001) and portal inflammation (r = −0.23, *p* = 0.002) (Figure 2).

Multivariate regression was performed after the correlation study, showing that GH as well as IGF1 values, adjusted for Tanner’s stage, sex and BMI, were negatively associated with HOMA-IR but above all with liver fibrosis (GH:→β = −2.3, SE 0.31, *p* = 0.001; IGF1:→β = −2.8, SE = 1.1, *p* = 0.001). Only GH was associated with TNF-a (β = −1.2, SE 0.12, *p* = 0.04) (Table 5).

## 5. Discussion

To our knowledge, our study is the first one performed on adolescents with MAFLD diagnosis sustained by liver biopsy, and it shows that serum GH levels are inversely correlated with the onset of liver fibrosis (r = −0.51, *p* = 0.0001). In patients with lower GH values (although within the normal range), we observed a more severe liver damage progression. Furthermore, the GH values appeared to be inversely correlated with lobular inflammation (r = −0.36, *p* = 0.02), which is a sign of chronic active inflammation of the liver induced by cytokines, and which activates hepatic stellate cells causing the release of collagen and the formation of fibrosis. These data are in accordance with the adult population and the literature on animal models [3], since changes in the GH/IGF1 axis are known to increase the risk of MAFLD [27]. Numerous studies conducted on animal models have shown that mice with liver-specific GH receptor knock-out show severe hepatic steatosis along with upregulation of inflammatory cytokines and increased transaminases, signaling hepatocellular damage [28]. This finding is also confirmed in clinical studies conducted on an adult population with GH deficiency, in which it was shown that patients have a higher prevalence of MAFLD, and that GH replacement betters the characteristics of this damage [29,30].

Another important finding that emerges from our study is that GH values are associated with alterations of lipid profile. Our adolescents with lower GH values have higher total cholesterol levels and significantly lower HDL levels (Table 5). In fact, GH suppresses hepatic lipogenesis “de novo”, stimulates lipolysis and increases lipid beta-oxidation by reducing the concentration of triglycerides in hepatocytes and altering the hepatic lipid production [31,32].

No less important in the pathogenesis of MAFLD appears to be the role of IGF1 and the IGF1/IGFBP-3 ratio. Hepatocytes do not express the IGF1 receptor, distinct from hepatic stellate cells and Kupffer cells [33]. In a recent study conducted in an adult population, patients with higher levels of serum IGF1 and with a higher IGF1/IGFBP-3 ratio had a lower risk of NASH, while patients with obesity with/without MAFLD always had lower serum IGF1 levels [33]. Furthermore, the IGF1/IGFBP-3 ratio significantly decreased with the increasing degree of fibrosis, i.e., with the progression towards an irreversible liver damage [28]. This finding is also confirmed in our pediatric study. In our adolescents, levels of IGF1 (r = −0.45, *p* = 0.0001) and of IGFBP-3 (r = −0.35, *p* = 0.0001) are inversely related to the degree of fibrosis (Table 4).

Regarding glucose homeostasis, our data are consistent with the known insulin-like effects of IGF1. In animals and humans, IGF1 suppresses glucagon and hepatic gluconeogenesis, increases peripheral glucose utilization, and suppresses lipolysis and protein breakdown [34]. These effects are mediated by the action of IGF1 on the insulin receptor but have also been shown to occur in part through signaling to the IGF1 receptor. In our patients, the reduced serum values of IGF1 are associated with elevated values of basal insulin and HOMA-IR (r = −0.32, *p* = 0.001) but also of total cholesterol (Table 4).

The role of obesity is another important point. Obesity is often associated with insulin resistance, glucose intolerance, and MAFLD. However, obesity is also associated with GH deficiency, resulting in decreased hepatic GH signaling and decreased hepatic synthesis of IGF1, two factors that may favor the development of steatosis. Many individuals with obesity have a (relatively physiological) impaired GH secretion that can be reversed by weight loss [35]. In our pediatric population, IGF1 levels are lower in patients with high BMI and with significant obesity (r = −0.15, *p* = 0.03). Although in the literature the total serum levels of IGF1 does not appear to decrease in obesity, and, on the contrary, levels of bioactive IGF1 are even slightly elevated when compared to normal weighting subjects, in populations affected by steatosis and steatohepatitis this picture changes. In a recent work, it was indeed demonstrated that IGF1 is strongly negatively associated both with MAFLD characteristics and with insulin resistance [3]. In the general population, adults with higher serum IGF1 levels and a higher serum IGF1/IGFBP-3 ratio have a lower risk of MAFLD [28]. In adults with obesity and/or MAFLD, serum IGF1 levels were lower in those with higher degrees of inflammation and higher stage of fibrosis, and the serum IGF1/IGFBP-3 ratio decreased with increasing fibrosis [36]. This data underlines how obesity affects the IGF-1 values, but once steatosis or, even more so, steatohepatitis is established, IGF1 levels are influenced by the presence of steatosis and/or fibrosis as well as by the glycemic profile, regardless of the BMI value. In accordance with these data, the use of GHRH analog to increase endogenous GH in adults with obesity and GH deficiency has been proposed, and it has shown a positive effect in reducing visceral fat, body fat mass and systemic inflammation [37,38].

We can conclude that our study demonstrated that even in the pediatric population, a reduction in GH signaling in the liver promotes the development of “de novo” hepatic lipogenesis, steatosis, inflammation and fibrosis. Thus, in the future it may be desirable to investigate whether recombinant GH administration may have a role also in adolescents with obesity and progressing MAFLD, in order to prevent the hepatic damage induced by steatosis, which appears to increasingly lead to the appearance of cirrhosis already in young adulthood.

## Figures and Tables

**Table 1:** Anthropometric and laboratory characteristics of population.

Variables	Mean (SD) or Median (25th–75th Centile)
Age, years	12.9 (2.1)
Sex (M/F)%	88/103 (46.2/53.8)
BMI, kg/sqm	28.5 (4.7)
WC, cm (IQR)	88.5 (81–99)
Uric acid, mg/dL	5.2 (1.6)
ALT, UI/L (IQR)	51 (31–78)
AST, UI/L (IQR)	39 (27–54)
GGT, UI/L (IQR)	21 (15–29)
Total-cholesterol, mg/dL (IQR)	158 (132–179)
HDL cholesterol, mg/dL(IQR)	43 (36–48)
Triglycerides, mg/dL (IQR)	138 (73–151)
Glucose, mg/dL (IQR)	82.9 (76–91)
Insulin, μUI/ML (IQR)	17.6 (10–28.3)
HOMA-IR	3.7 (3.4)
DBP, mmHg (IQR)	68 (60–74)
SBP, mmHg (IQR)	110 (101–118)
GH, ng/mL (IQR)	7.8 (5.8–9)
IGF1, ng/mL (IQR)	266 (198–345)
IGFBP-3, µg/mL (IQR)	2.34 (1.5–3.2)
TNF-a (IQR), pg/ml	51 (7.5–77.3)
IL-6 (IQR), pg/mL	29 (19–33.5)

BMI = Body Mass Index; WC = Waist Circumference; AST = aspartate aminotransferase; ALT = alanine aminotransferase; GGT = gamma-glutamyl transferase; HDL = High Density Lipoprotein; HOMA-IR = HOmeostasis Model Assessment-estimated Insulin Resistance; DBP = Diastolic Blood Pressure; SBP = Systolic Blood Pressure; GH = Growth Hormone; IGF1 = Insulin-like Growth Factor; IGFBP-3 = Insulin-like Growth Factor-Binding Protein 3; TNF-a = Tumor Necrosis Factor; IL-6 = interleukine-6.

**Table 2:** Anthropometric and laboratory characteristics in the sample divided by Tanner’s stage (and approximate age) and gender.

	Tanner’s Stage I–II–III (Approximatively 12–15 Years)			Tanner’s Stage IV–V (Approximatively > 15 Years)			Tanner’s Stage: I–II–III vs. IV–V *p* Values
	All (151)	F (85)	M (66)	All (40)	F (18)	M (22)	
Age, years	13.5 (1.33)	12.9 (1.3)	12.6(1.3)	15.9 (1.1)	15.9 (1.3)	16 (0.9)	0.0001
BMI, kg/sqm	27.1 (4.2)	27 (24–29)	27.1 (24–29.5)	29.8 (5.6)	30 (25–33)	28.8 (25–31)	0.01
WC, cm (IQR)	84 (78–92)	83 (78–92)	83 (77–90.5)	88 (78–97)	87 (76–96)	88 (81–97)	0.04
Uric acid, mg/dL	5.2 (1.6)	5.4 (1.6)	5 (1.5)	5.7 (1.6)	5.3 (1.9)	6 (1.2)	0.14
ALT, UI/L (IQR)	59.7 (32–75.5)	63 (33–78)	55 (31–73)	73 (28–96)	85 (32–130)	65 (28–78)	0.08
AST, UI/L (IQR)	43 (27–52.5)	44 (30–56)	41.5 (26–49)	45 (25–58)	57 (24–67)	39.5 (25–44.7)	0.65
GGT, UI/L (IQR)	23.6 (15–27)	25 (15–30)	22 (14–25)	32 (14–39)	39 (31–18)	26 (13–28)	0.01
Total-cholesterol, mg/dL (IQR)	158 (132–178)	165 (145–189)	149 (123–169)	161 (131–180)	173 (131–216)	151 (132–165)	0.66
HDL, mg/dL (IQR)	46 (38–48.5)	44.6 (39–49)	47 (36–48)	45 (36–48)	46 (36–53)	45 (35–44)	0.82
Triglycerides, mg/dL (IQR)	104 (69–117)	109 (97–122)	99 (59–110)	126 (89–168)	125 (90–170)	127 (89–163)	0.05
Glucose, mg/dL (IQR)	83.6 (76–90)	83 (76–89)	84 (77–93)	80 (76–86)	85 (77–92)	78 (71–83)	0.13
Insulin, μUI/ML (IQR)	17 (10–24)	19 (10–25.5)	16 (10–21)	18(10–24)	22 (11–29)	15 (10–19)	0.71
HOMA-IR (IQR)	3.7 (2–4.9)	3.9 (2.2–4.9)	3.4 (2–5)	3.8 (2–4.5)	4.9 (2.8–6.9)	3.1 (1.8–4)	0.81
DBP, mmHg (IQR)	67 (60–74)	67 (59–79)	67.5 (61–73)	66 (60–73)	70 (68–75)	63 (58–68)	0.49
SBP, mmHg (IQR)	110 (101–118)	110.5 (102–118)	111 (102–115)	111.5 (103–120)	110 (105–118)	112 (102–120)	0.62
TNF-a	40 (7–47)	41 (7–22.5)	36.5(12–59)	55.2 (43–89)	44.5 (30–49)	41 (33–78)	0.06
IL-6	26 (18–31.2)	24 (19–29.5)	27 (18.5–32)	36.5 (25–50)	25 (21–35)	39 (26–49)	0.13
GH, ng/mL (IQR)	10.2 (8–11)	10.5(3.2)	10 (8–11)	10 (8–11)	8.1 (7.5–11)	10.4 (2–11.3)	0.67
IGF1, ng/mL(IQR)	272 (198–345)	267 (198–305)	279 (208–347)	268 (198–345)	233 (164–305)	395 (125–205)	0.81
IGFBP-3, µg/mL (IQR)	2.4 (1.5–3.2)	2.4 (1.5–3.2)	2.4 (1.5–3.2)	2.2 (1.5–2.8)	2.2 (1.4–2.6)	2.3 (1.6–2.9)	0.5

**Table 3:** Histological characteristics of the population. NAS score distribution.

Histologic Characteristic	Number of Patients	Percentage
**Steatosis**		
**0**	16	8.4%
**1**	64	33.7%
**2**	72	37.9%
**3**	38	20%
**Portal inflammation**		
**0**	19	10%
**1**	139	73.2%
**2**	32	16.8%
**Lobular inflammation**		
**0**	73	38.6%
**1**	100	52.9%
**2**	16	8.5%
**Ballooning**		
**0**	99	52.1%
**1**	62	32.6%
**2**	29	15.3%
**Fibrosis**		
**0**	57	30%
**1**	108	56.8%
**2**	18	9.5%
**3**	7	3.7%
**NAS**		
**0**	13	6.8%
**1**	11	5.8%
**2**	43	22.6%
**3**	34	17.9%
**4**	39	20.5%
**5**	23	12.6%
**6**	24	12.6%
**7**	3	1.6%

NAS = scoring system for non-alcoholic steatohepatitis.

**Table 4:** Characteristics of the population according to the diagnosis of steatohepatitis.

	Non-Steatohepatitis (N 102)	Steatohepatitis (N 89)	*p*
Age, years	12.9 (1.6)	13.1 (1.8)	0.40
Sex, (F/M)	57/45	46/43	0.79
BMI, kg/sqm	26.8 (4.2)	28.3 (4.4)	0.01
WC, cm (IQR)	85.9 (72–95)	90 (80–99)	0.02
Uric acid, mg/dL	5.4 (1.6)	6 (1.4)	0.01
ALT, UI/L (IQR)	49 (24–66)	75 (39–90)	0.001
AST, UI/L (IQR)	37 (25–45)	50 (33–62)	0.001
GGT, UI/L (IQR)	21 (13–24)	30 (19–38)	0.04
Total-cholesterol, mg/dL (IQR)	158 (93–175)	161 (78–183)	0.22
HDL, mg/dL (IQR)	44 (37–49)	47 (35–48)	0.70
Triglycerides, mg/dL (IQR)	100 (73–138)	127 (74–146)	0.04
Glucose, mg/dL (IQR)	80 (75–86)	89 (76–96)	0.03
Insulin, μUI/ML (IQR)	17 (10–24)	24 (18–32)	0.02
HOMA-IR (IQR)	3.5 (2.2–4.5)	4.6 (3.2–6.2)	0.001
DBP, mmHg (IQR)	65 (58–72)	68 (61–75)	0.08
SBP, mmHg (IQR)	110 (101–118)	114 (103–121)	0.31
TNF-a, pg/mL	44 (7.5–44)	72 (12–72)	0.02
IL-6, pg/mL	25 (22–35.5)	35 (15–39)	0.048
GH, ng/mL (IQR)	7.5 (6–8.2)	5.4 (4–6.8)	0.001
IGF1, ng/mL(IQR)	284 (208–345)	245 (150–310)	0.01
IGFBP-3, µg/mL (IQR)	2.6 (1.6–3.4)	2 (1.4–2.8)	0.02

BMI = Body Mass Index; WC = Waist Circumference; AST = aspartate aminotransferase; ALT = alanine aminotransferase; GGT = gamma-glutamyl transferase; HDL = High Density Lipoprotein; HOMA-IR = HOmeostasis Model Assessment-estimated Insulin Resistance; DBP = Diastolic Blood Pressure; SBP = Systolic Blood Pressure; GH = Growth Hormone; IGF1 = Insulin-like Growth Factor 1; IGFBP-3 = Insulin-like Growth Factor-Binding P Correlation of GH, IGF1 and IGFBP-3 with metabolic parameters and histology.

**Table 5:** Logistic regression analysis of GH and IGF1.

Logistic Regression Analysis *	Standardized Beta Coefficients	SE	*p* Value
**GH, ng/mL**			
Total Cholesterol	0.13	0.04	**0.003**
HDL-Cholesterol	−0.17	0.06	**0.008**
Triglycerides	−0.01	0.02	0.37
Glucose	1.7	0.02	**0.002**
Insulin	0.03	0.04	0.45
HOMA-IR	−0.33	0.2	0.09
Fibrosis	−2.3	0.31	**0.001**
Portal Inflammation	0.56	0.51	0.41
Lobular Inflammation	−0.36	0.34	0.28
TNF-a	−1.2	0.12	**0.04**
**IGF1, ng/mL**			
Total Cholesterol	0.41	1.3	0.75
HDL-Cholesterol	0.68	1.97	0.72
Triglycerides	−0.07	0.35	0.83
Glucose	−0.69	0.71	0.32
Insulin	3.8	1.5	**0.01**
HOMA-IR	−2.7	1.7	**0.001**
Fibrosis	−2.8	1.1	**0.001**
Portal Inflammation	−1.3	1.7	0.09
Lobular Inflammation	−1.0	1.1	0.42
TNF-a	−0.78	0.34	0.25
**IGFBP-3, µg/mL**			
Total Cholesterol	0.01	0.01	0.46
HDL-Cholesterol	−0.02	0.03	0.21
Triglycerides	0.01	0.04	0.69
Glucose	−0.01	0.07	0.16
Insulin	−0.02	0.016	0.94
HOMA-IR	−0.09	0.07	0.20
Fibrosis	−0.02	0.04	0.45
Portal Inflammation	0.41	0.54	0.35
Lobular Inflammation	−0.01	0.9	0.74
TNF-a	−0.11	0.02	0.85

* Adjusted for BMI, WC and gender. HDL = High Density Lipoprotein; HOMA-IR = HOmeostasis Model Assessment-estimated Insulin Resistance. The *p* values in bold are the significant ones.

## Data Availability

The data presented in this study are available in the main article.

## Abbreviations

NALFD = Non-alcoholic fatty liver disease; NASH = non-alcoholic steatohepatitis; BMI = body mass index; WC = waist circumference; GH = growth hormone; IGF1 = insulin-like growth factor; IGFBP-3 = Insulin-like growth factor-binding protein 3; AST = aspartate aminotransferase; ALT = alanine aminotransferase; HOMA-IR = homeostatic model assessment for insulin resistance; GGT = gamma-glutamyl-transferase; IQR = interquartile intervals; LDL = low density lipoprotein-cholesterol; HDL = high density lipoprotein-cholesterol; SD = standard deviation.

## References

[1] Liang S., Yu Z., Song X., Wang Y., Li M., Xue J. (2018). Reduced Growth Hormone Secretion is Associated with Nonalcoholic Fatty Liver Disease in Obese Children. Horm. Metab. Res..

[2] Cabrera D., Cabello-Verrugio C., Solís N., San Martín D., Cofré C., Pizarro M., Arab J.P., Abrigo J., Campos F., Irigoyen B. (2018). Somatotropic Axis Dysfunction in Non-Alcoholic Fatty Liver Disease: Beneficial Hepatic and Systemic Effects of Hormone Supplementation. Int. J. Mol. Sci..

[3] Stanley T.L., Fourman L.T., Zheng I., McClure C.M., Feldpausch M.N., Torriani M., Corey K.E., Chung R.T., Lee H., Kleiner D.E. (2021). Relationship of IGF-1 and IGF-Binding Proteins to Disease Severity and Glycemia in Nonalcoholic Fatty Liver Disease. J. Clin. Endocrinol. Metab..

[4] Ichikawa T., Nakao K., Hamasaki K., Furukawa R., Tsuruta S., Ueda Y., Taura N., Shibata H., Fujimoto M., Toriyama K. (2007). Role of growth hormone, insulin-like growth factor 1 and insulin-like growth factor-binding protein 3 in development of non-alcoholic fatty liver disease. Hepatol. Int..

[5] Clemmons D.R. (2004). The relative roles of growth hormone and IGF-1 in controlling insulin sensitivity. J. Clin. Investig..

[6] Fan Y., Menon R.K., Cohen P., Hwang D., Clemens T., DiGirolamo D.J., Kopchick J.J., Le Roith D., Trucco M., Sperling M.A. (2009). Liver-specific deletion of the growth hormone receptor reveals essential role of growth hormone signaling in hepatic lipid metabolism. J. Biol. Chem..

[7] Fusco A., Miele L., D’Uonnolo A., Forgione A., Riccardi L., Cefalo C., Barini A., Bianchi A., Giampietro A., Cimino V. (2012). Nonalcoholic fatty liver disease is associated with increased GHBP and reduced GH/IGF-I levels. Clin. Endocrinol..

[8] Bredella M.A., Torriani M., Thomas B.J., Ghomi R.H., Brick D.J., Gerweck A.V., Miller K.K. (2009). Peak growth hormone-releasing hormone-arginine-stimulated growth hormone is inversely associated with intramyocellular and intrahepatic lipid content in premenopausal women with obesity. J. Clin. Endocrinol. Metab..

[9] Clemmons D.R. (2018). Role of IGF-binding proteins in regulating IGF responses to changes in metabolism. J. Mol. Endocrinol..

[10] Martinez-Castillo M., Rosique-Oramas D., Medina-Avila Z., Perez-Hernandez J.L., Higuera-De la Tijera F., Santana-Vargas D., Montalvo-Jave E.E., Sanchez-Avila F., Torre A., Kershenobich D. (2020). Differential production of insulin-like growth factor-binding proteins in liver fibrosis progression. Mol. Cell. Biochem..

[11] Petaja E.M., Zhou Y., Havana M., Hakkarainen A., Lundbom N., Ihalainen J., Yki-Jarvinen H. (2016). Phosphorylated IGFBP 1 as a non-invasive predictor of liver fat in NAFLD. Sci. Rep..

[12] Kotronen A., Lewitt M., Hall K., Brismar K., Yki-Jarvinen H. (2008). Insulin-like growth factor binding protein 1 as a novel specific marker of hepatic insulin sensitivity. J. Clin. Endocrinol. Metab..

[13] Fahlbusch P., Knebel B., Horbelt T., Barbosa D.M., Nikolic A., Jacob S., Al-Hasani H., Van de Velde F., Van Nieuwenhove Y., Muller-Wieland D. (2020). Physiological Disturbance in Fatty Liver Energy Metabolism Converges on IGFBP2 Abundance and Regulation in Mice and Men. Int. J. Mol. Sci..

[14] Dali-Youcef N., Vix M., Costantino F., El-Saghire H., Lhermitte B., Callari C., D’Agostino J., Perretta S., Paveliu S., Gualtierotti M. (2019). Interleukin-32 Contributes to Human Nonalcoholic Fatty Liver Disease and Insulin Resistance. Hepatol. Commun..

[15] Min H.K., Maruyama H., Jang B.K., Shimada M., Mirshahi F., Ren S., Oh Y., Puri P., Sanyal A.J. (2016). Suppression of IGF binding protein-3 by palmitate promotes hepatic inflammatory responses. FASEB J..

[16] Lopez-Bermejo A., Khosravi J., Fernandez-Real J.M., Hwa V., Pratt K.L., Casamitjana R., Garcia-Gil M.M., Rosenfeld R.G., Ricart W. (2006). Insulin resistance is associated with increased serum concentration of IGF-binding protein-related protein 1 (IGFBP-rP1/MAC25). Diabetes.

[17] Yan H., Li T., Wang Y., Li H., Xu J., Lu X. (2019). Insulin-like growth factor binding protein 7 accelerates hepatic steatosis and insulin resistance in non-alcoholic fatty liver disease. Clin. Exp. Pharmacol. Physiol..

[18] Takahashi Y., Iida K., Takahashi K., Yoshioka S., Fukuoka H., Takeno R., Imanaka M., Nishizawa H., Takahashi M., Seo Y. (2007). Growth hormone reverses nonalcoholic steatohepatitis in a patient with adult growth hormone deficiency. Gastroenterology.

[19] Sobrevals L., Rodriguez C., Romero-Trevejo J.L., Gondi G., Monreal I., Paneda A., Juanarena N., Arcelus S., Razquin N., Guembe L. (2010). Insulin-like growth factor I gene transfer to cirrhotic liver induces fibrolysis and reduces fibrogenesis leading to cirrhosis reversion in rats. Hepatology.

[20] Vajro P., Lenta S., Socha P., Dhawan A., McKiernan P., Baumann U., Durmaz O., Lacaille F., McLin V., Nobili V. (2012). Diagnosis of nonalcoholic fatty liver disease in children and adolescents: Position paper of the ESPGHAN Hepatology Committee. J. Pediatr. Gastroenterol. Nutr..

[21] Feldstein A.E., Charatcharoenwitthaya P., Treeprasertsuk S., Benson J.T., Enders F.B., Angulo P. (2009). The natural history of non-alcoholic fatty liver disease in children: A follow-up study for up to 20 years. Gut.

[22] Rosenfeld R.G., Albertsson-Wikland K., Cassorla F., Frasier S.D., Hasegawa Y., Hintz R.L., LaFranchi S., Lippe B., Loriaux L., Melmed S. (1995). Diagnostic controversy: The diagnosis of childhood growth hormone deficiency revisited. J. Clin. Endocrinol. Metab..

[23] Linea Guida Clinica Sulla Diagnosi del Deficit di GH. https://www.ospedalebambinogesu.it/.

[24] Conwell L.S., Trost S.G., Brown W.J., Batch J.A. (2004). Indexes of insulin resistance and secretion in obese children and adolescents: A validation study. Diabetes Care.

[25] Brunt E.M., Kleiner D.E., Wilson L.A., Belt P., Neuschwander-Tetri B.A., NASH Clinical Research Network (CRN) (2011). Nonalcoholic fatty liver disease (NAFLD) activity score and the histopathologic diagnosis in NAFLD: Distinct clinicopathologic meanings. Hepatology.

[26] Santiago-Rolón A., Purcell D., Rosado K., Toro D.H. (2015). A comparison of brunt’s criteria, the non-alcoholic fatty liver disease activity score (NAS), and a proposed NAS scoring that includes fibrosis in non-alcoholic fatty liver disease staging. Puerto Rico Health Sci. J..

[27] Rufinatscha K., Ress C., Folie S., Haas S., Salzmann K., Moser P., Dobner J., Weiss G., Iruzubieta P., Arias-Loste M.T. (2018). Metabolic effects of reduced growth hormone action in fatty liver disease. Hepatol. Int..

[28] Dichtel L.E., Corey K.E., Misdraji J., Bredella M.A., Schorr M., Osganian S.A., Young B.J., Sung J.C., Miller K.K. (2017). The Association Between IGF-1 Levels and the Histologic Severity of Nonalcoholic Fatty Liver Disease. Clin. Transl. Gastroenterol..

[29] Dichtel L.E., Cordoba-Chacon J., Kineman R.D. (2022). Growth Hormone and Insulin-Like Growth Factor 1 Regulation of Nonalcoholic Fatty Liver Disease. J. Clin. Endocrinol. Metab..

[30] Osganian S.A., Subudhi S., Masia R., Drescher H.K., Bartsch L.M., Chicote M.L., Chung R.T., Gee D.W., Witkowski E.R., Bredella M.A. (2022). Expression of IGF-1 receptor and GH receptor in hepatic tissue of patients with nonalcoholic fatty liver disease and nonalcoholic steatohepatitis. Growth Horm. IGF Res..

[31] Mauras N., O’Brien K.O., Welch S., Rini A., Helgeson K., Vieira N.E., Yergey A.L. (2000). Insulin-like growth factor I and growth hormone (GH) treatment in GH-deficient humans: Differential effects on protein, glucose, lipid, and calcium metabolism. J. Clin. Endocrinol. Metab..

[32] Lambert J.E., Ramos-Roman M.A., Browning J.D., Parks E.J. (2014). Increased de novo lipogenesis is a distinct characteristic of individuals with nonalcoholic fatty liver disease. Gastroenterology.

[33] Cordoba-Chacon J., Sarmento-Cabral A., Del Rio-Moreno M., Diaz-Ruiz A., Subbaiah P.V., Kineman R.D. (2018). Adult-Onset Hepatocyte GH Resistance Promotes NASH in Male Mice, Without Severe Systemic Metabolic Dysfunction. Endocrinology.

[34] Pan C.S., Weiss J.J., Fourman L.T., Buckless C., Branch K.L., Lee H., Torriani M., Misra M., Stanley T.L. (2021). Effect of recombinant human growth hormone on liver fat content in young adults with nonalcoholic fatty liver disease. Clin. Endocrinol..

[35] Hjelholt A., Høgild M., Bak A.M., Arlien-Søborg M.C., Bæk A., Jessen N., Richelsen B., Pedersen S.B., Møller N., Jørgensen J.O.L. (2020). Growth Hormone and Obesity. Endocrinol. Metab. Clin. N. Am..

[36] Hribal M.L., Procopio T., Petta S., Sciacqua A., Grimaudo S., Pipitone R.M., Perticone F., Sesti G. (2013). Insulin-like growth factor-I, inflammatory proteins, and fibrosis in subjects with nonalcoholic fatty liver disease. J. Clin. Endocrinol. Metab..

[37] Attallah H., Friedlander A.L., Hoffman A.R. (2006). Visceral obesity, impaired glucose tolerance, metabolic syndrome, and growth hormone therapy. Growth Horm. IGF Res..

[38] Bredella M.A., Gerweck A.V., Lin E., Landa M.G., Torriani M., Schoenfeld D.A., Hemphill L.C., Miller K.K. (2013). Effects of GH on body composition and cardiovascular risk markers in young men with abdominal obesity. J. Clin. Endocrinol. Metab..

